# Altered or Impaired Immune Response to Hepatitis B Vaccine in WNIN/GR-Ob Rat: An Obese Rat Model with Impaired Glucose Tolerance

**DOI:** 10.5402/2011/980105

**Published:** 2011-08-10

**Authors:** Prathibha Bandaru, Hemalatha Rajkumar, Giridharan Nappanveettil

**Affiliations:** ^1^Department of Microbiology, National Institute of Nutrition, Jamai Osmania, Hyderabad 500 604, India; ^2^National Centre for Laboratory Animal Sciences, National Institute of Nutrition, Indian Council of Medical Research, Jamai Osmania, Andhra Pradesh, Hyderabad 500 604, India

## Abstract

Obesity is shown to increase the incidence and severity of infectious diseases and individuals seem to exhibit poor antibody response to vaccination due to several inherent immune defects. With the increasing prevalence of impaired glucose tolerance (IGT) seen in obese individuals, the present study was aimed to investigate the basal immune response and immune response upon Hepatitis B vaccination (HBV) in an obese rat model WNIN/GR-Ob with impaired glucose tolerance (IGT). Decreased proportions of splenic CD4^+^ T helper cells and CD3^+^ T cells were observed in obese animals compared to lean animals. Upon HBV, obese animals showed reduced cell-mediated immunity and humoral immunity in terms of splenic lymphocyte proliferative response to Concanavalin A (Con A) and Hepatitis B surface antigen (HBsAg) and HBsAg-specific IgG response. Innate immunity as assessed in terms of Tumor Necrosis Factor **α** (TNF **α**) and Nitric oxide (NO) production by peritoneal macrophages upon HBV was low and unchanged, respectively, in obese animals. Thus long-term immunological memory is impaired or altered upon HBV.

## 1. Introduction

Obesity is often associated with increased risk of degenerative diseases like type 2 diabetes, cardiovascular disease (CVD), and cancer [1]. However, clinically a positive correlation between body weight index and incidence of nosocomial infections was also observed [2]. Increased infections were reported in genetically obese and diet-induced obese rodent models. For example, Zucker obese rats showed an increased susceptibility to Candida albicans infections [3], while Ob/Ob mice displayed impaired immune response to Listeria monocytogenes and Candida albicans [4]. In diet-induced obese (DIO) rodent models, a poor response to Porphyromonas gingivalis infection were observed and infection with influenza virus seemed to cause high mortality rate in mice [5, 6].

In obese individuals reduction in lymphocyte numbers and reduced responsiveness to mitogen were observed [7, 8]. Defects in specific immunity such as reduced lymphocyte numbers in spleen, thymus, and peripheral blood have been reported in Ob/Ob and db/db mice and fa/fa Zucker rats [9]. Diminished responsiveness to mitogen as well as cytotoxic activity was also seen in these animals [9]. Same was true in DIO models where reduced lymphocyte response and altered cytokine secretion were observed [10]. Poor antibody response was reported in obese individuals and overweight children's against vaccination [11–13]. Furthermore, reduced lymphocyte function upon HBV was observed in WNIN/Ob obese rat [14]. 

In recent years, increased prevalence of obesity in both the developed and developing countries is accompanied by a parallel rise in the incidence of impaired glucose tolerance (IGT) both in adults and children. In this context, an attempt was made to study the immune response in an obese rat model with IGT. At National Institute of Nutrition, we have a spontaneously mutated obese rat model, namely, WNIN/GR-Ob, which exhibits the whole gamut of characteristics of metabolic syndrome [15–18]. In general, the fasting blood glucose levels of these obese animals are 90–100 mg/dL, whereas oral lavage with glucose at a dose of 250 mg/100 g body weight, the 1 h and 2 h blood glucose levels, were beyond 140 mg/dL suggesting that the animals exhibited impaired glucose tolerance [19]. These animals also develop kidney dysfunction, tumors, and opportunistic infections as they cross one year of age and their life span is short (1.5 years versus 3 years of normal rats) suggesting an altered or impaired immune function. In the present study, basal immune response and immune response to Hepatitis B vaccine (HBV) was studied in WNIN/GR-Ob obese rat with IGT.

## 2. Materials and Methods

### 2.1. Studies on Basic Immune Response

#### 2.1.1. Animals

90-day old 8 female rats of lean (+/+) and obese (−/−) phenotypes were obtained from the stock colony of NCLAS and were fed on standard pellet diet. Animals were housed individually in the animal facility with proper temperature (22 ± 2°C), humidity (50–55%), and light control (12 h light and 12 h darkness) and were provided with ad libitum rat chow and water. The study design had the approval of the Institutional Animal Ethical Committee (IAEC). Blood was collected through retroorbital sinus and the animals were sacrificed to perform the following immune parameters.

#### 2.1.2. Body and Spleen Weights

Body weights of the animals were taken. Animals were euthanized using CO_2_ inhalation and spleen was removed aseptically and weighed. The spleen weight was then normalized to gram body weight.

#### 2.1.3. Splenic Lymphocyte Proliferation Assay

Lymphocyte proliferation assay was performed as previously described [20]. Briefly, splenocytes were dissociated by using a stainless steel screen and adjusted to 1 × 10^9^ cells/L RPMI 1640 medium supplemented with 40,000 *μ*g/L gentamycin and 5% FBS (Sigma-Aldrich). 200 *μ*L of the cell suspension was added to each well in a 96-well polystyrene plate and incubated for 48 h at 37°C, 5% CO_2_ in the absence and presence of 2.5 *μ*g/mL of Con A (Himedia). After two days the cultures were pulsed with 0.5 *μ*ci of [^3^H] thymidine (specific activity 240 Bq/mmole; BRIT; Mumbai, India). Twenty-four hours later, the cells were harvested onto a glass fiber filter. Radioactivity was then measured using a liquid scintillation counter (Packard Tri-Carb Liquid Scintillation Counter) after the filters had been kept overnight at room temperature. Each test was performed in triplicates. Splenic lymphocyte proliferative response was expressed in terms of CPM of Con A stimulated (T)/CPM of unstimulated cells (C).

#### 2.1.4. Splenic Lymphocyte Subpopulation Measurement

The splenic lymphocyte subpopulation was measured by immunofluorescent antibody staining procedure using flow cytometry [21] (Partec PAS). Briefly, an aliquot (1-2 millions) of freshly isolated cells was washed with FACS buffer (PBS with 5% FBS and 0.1% sodium azide) and was stained with the following antibodies: fluorescein isothiocyanate- (FITC-) conjugated anti-rat CD4 (clone OX-35); phycoerythrin (PE) anti-rat CD3 (clone G4.18); phycoerythrin (PE) anti-rat CD8a (clone OX-8); purified anti-rat CD45RA; IgM and fluorescein-isothiocyanate (FITC-) conjugated rat anti-mouse IgG1 antibody. All antibodies were procured from BD Biosciences. Cells were incubated with antibody for 30 min at 4°C and then washed three times with FACS buffer. Cells were analyzed with a flow cytometer. The samples were gated using forward versus 90-degree light scatter to exclude granulocytes and monocytes from the splenocytes population. For each test sample, 20,000 cells were analyzed.

#### 2.1.5. Estimation of Serum Immunoglobulins

Total IgG and IgM levels were measured in serum using commercially available ELISA kit from Bethyl laboratories Inc. IgG sensitivity was >7.8 ng/mL whereas that of IgM was >31.25 ng/mL.

#### 2.1.6. Studies on Immune Response upon Hepatitis B Vaccination

Fourteen (*n* = 14) 90-day-old obese and lean female animals of WNIN/GR-Ob strain were obtained from National Centre for Laboratory Animal Sciences (NCLAS). Six (*n* = 6) out of these fourteen were grouped as controls or unvaccinated animals and were given Phosphate Buffered Saline (PBS), whereas the remaining eight animals (*n* = 8) were grouped as vaccinated and were administered Hepatitis B vaccine. 4 *μ*g of Hepatitis B vaccine (Shanvac from Shantha Biotech, Hyderabad, India) was administered intramuscularly and a booster dose was injected one month after the first dose [22]. One week after the booster dose, blood was collected from the retroorbital sinus vein and the animals were sacrificed to perform the following immune parameters.

#### 2.1.7. Antigen Specific Antibody Production

Presence of antibodies to HBsAg (a kind gift from Shantha Biotech) in the serum was determined by ELISA in flat bottom 96-well plates (NUNC-Immuno Plate (polySorp)) [23]. Sera of unvaccinated lean and obese animals were taken as negative controls. Briefly, the plates were coated with 1 *μ*g/mL (100 *μ*L/well) HBsAg in bicarbonate buffer (0.1 M, pH 9.6) and incubated overnight at 4°C. Plates were washed with a 0.1% solution of Tween-20 in PBS between all steps in a Labsystems Microplate Washer (Finland). After coating and washing, the plates were incubated with blocking buffer (PBS with 0.1% Tween-20 and 2% milk powder) for 1 h at 37°C. Subsequently, plates were washed and serum at a dilution of 1 : 5000 was added to the wells. After 1 h incubation at room temperature (RT), the plates were washed and anti-rat IgG peroxidase conjugate (100 *μ*L of 1 : 5000 dilution) (Sigma-Aldrich) was added and incubated for 1 h. Plates were washed again and O-Phenylene diamine dihydrochloride (OPD) 0.3 mg/mL (Sigma-Aldrich) plus 0.006% H_2_O_2_ in 0.15 M citrate buffer, pH 5.0, was added. The reaction was stopped using H_2_SO_4_ and the plates were read for absorbance at 492 nm in Labsystems ELISA microplate reader.

#### 2.1.8. Isolation and Culture of Peritoneal Macrophages

Peritoneal macrophages were obtained by washing the peritoneal cavity with 15 mL of RPMI 1640 medium [24]. Washed-out medium was centrifuged at 1200 g for 10 min at 4°C. The pellet containing macrophages was suspended in RPMI 1640 medium enriched with fetal bovine serum (5%) and gentamycin. Cell viability was determined by the trypan blue exclusion test and was >95%. Macrophage-rich cultures were obtained after 2 h incubation (37°C, 5% CO_2_) of 1 × 10^6^ cells/mL in 24-well polystyrene culture plates. Removal of nonadherent cells was done by washing the plate twice with RPMI 1640. The resulting adherent population consisted of >95% peritoneal macrophages. The peritoneal macrophage cultures with and without Lipopolysaccharide (LPS-1 *μ*g/mL) were incubated for 48 h. The culture supernatants was then taken for the estimation of NO and TNF *α* release.

#### 2.1.9. Nitric Oxide and TNF *α* Production by Peritoneal Macrophages

Nitrite (NO_2_^−^) which is the stable end product of NO was measured by a colorimetric assay using griess reagent. Nitrite concentration was calculated from NaNO_2_ standard curve [25]. The culture supernatant was collected and stored at −80°C until further analysis of TNF *α* by ELISA (R&D systems).

#### 2.1.10. Splenic Lymphocyte Proliferation Assay to HBsAg

Splenic lymphocyte proliferation assay in the presence of hepatitis B surface antigen at a final concentration of 2.5 *μ*g/mL was performed as described previously [22].

#### 2.1.11. Determination of Cytokines from Splenocyte Culture Supernatant

1 × 10^6^ splenocytes per mL were added to each well in a 24-well polystyrene plate and incubated for 24 h at 37°C, 5% CO_2_ in the absence or presence of 2.5 *μ*g/mL of Concanavalin A. After 24 h the culture supernatants were collected and stored at −80°C until further analysis of IL 2 and IL 4 by ELISA (R&D systems) [26].

#### 2.1.12. Statistics

Statistical analysis was conducted using SPSS 11.0 software. All data were reported as mean ± SE. The difference in the basal immune response between obese and lean phenotypes was analyzed by Student's *t*-test. Immune response to Hepatitis B vaccination between vaccinated and unvaccinated animals of both phenotypes was analyzed by one way analysis of variance (ANOVA). The significant differences between groups were identified by least significant difference at *P* < 0.05.

## 3. Results

### 3.1. Basal Immune Response

The body weight of obese animals (400 ± 3.9 g) was significantly higher (209 ± 5.3 g) whereas the spleen weight/g body weight was significantly lower compared to lean females. The obese animals showed significant decrease in CD4^+^ helper T cells, and CD3^+^ T cells compared to lean animals, whereas the CD8^+^ cytotoxic T cells, B cells and splenic lymphocyte proliferative response to mitogen were comparable between obese and lean animals. However, the serum IgG and IgM levels were higher in obese females compared to lean animals (Table 1).

### 3.2. Immune Response upon Vaccination

#### 3.2.1. HBsAg Specific IgG Response

**Figure 1:**
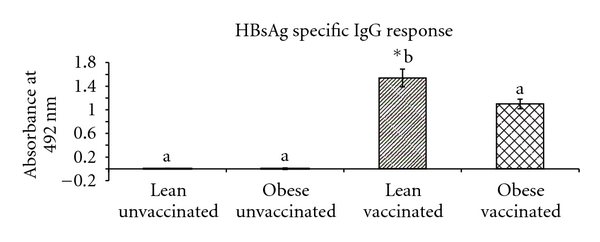
HBsAg-specific IgG response to Hepatitis B vaccine in 90-day-old WNIN/GROb lean and obese rats. Values are in Mean ± SE; *P < 0.05 (significant difference between unvaccinated and vaccinated groups of lean and obese rats).

Both the obese and lean animals responded to vaccine by the production of HBsAg specific IgG antibody response one week after the booster dose. However the antibody response was significantly low in obese vaccinated as compared to lean vaccinated (Figure 1). 

#### 3.2.2. Nitric Oxide (NO) and Tumor Necrosis Factor Alpha (TNF *α*) Production by Peritoneal Macrophages

Nitrate production by macrophages when stimulated with LPS was significantly higher in obese unvaccinated animals compared to lean unvaccinated. However, there was increased nitrate production in lean vaccinated but not in obese vaccinated. LPS stimulated TNF *α* production by peritoneal macrophages was significantly low in obese vaccinated compared to lean vaccinated (Table 2).

#### 3.2.3. Splenic Lymphocyte Proliferation

**Figure 2:**
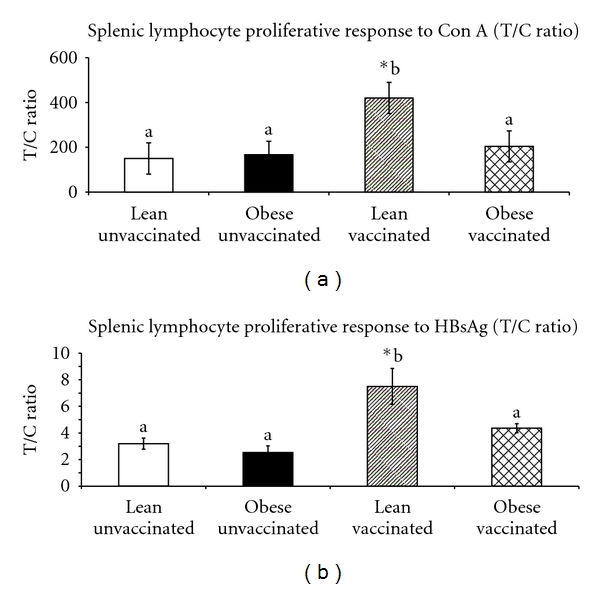
Splenic lymphocyte proliferative response (T/C ratio) to Con A (a) and HBsAg (b) by incorporation of 3H thymidine in 90-days-old WNIN/GR-Ob lean and obese vaccinated animals. Values are Mean ± SE; *P < 0.05 (significant difference between unvaccinated and vaccinated groups of lean and obese rats).

 In obese and lean unvaccinated animals the splenic lymphocyte proliferative response to mitogen was comparable. However, vaccination induced a significant increase in the splenic lymphocyte proliferative response to Con A and HBsAg in lean vaccinated compared to obese vaccinated animals (Figures 2(a) and 2(b)).

#### 3.2.4. Cytokine Production by Splenocytes

IL4 was not detectable in both stimulated and unstimulated splenocytes culture supernatant, whereas IL2 was detectable in splenocytes culture supernatant only. Con A stimulated IL2 production was comparable between obese and lean unvaccinated and vaccinated animals (Table 2).

## 4. Discussion

Globally the incidence of IGT has been increasing in obese children and adults in both the developed and developing countries [27, 28]. IGT is a prediabetic state and is associated with increased risk of cardiovascular diseases [29]. Rodent models have provided useful insights into the pathophysiology of obesity and obesity-related complications such as type 2 diabetes. However, such models are of substantial value in studying the complications of diabetes and the effect of prolonged hyperglycemia, but they may be less suitable for studying the milder changes that occur in the preclinical stages of type 2 diabetes, that is, prediabetes and the factors involved in the progression to overt fasting hyperglycemia and loss of glucose tolerance. Thus, WNIN/GR-Ob obese rat model with impaired glucose tolerance can be used as a suitable model to study the milder changes that are involved in the preclinical stages of type 2 diabetes in obese condition.

Obese rats showed significantly low spleen weights, CD4^+^ helper T cells and CD3^+^ T cells which is in agreement with the findings observed in fa/fa Zucker rats [30, 31]. The splenic lymphocyte mitogenic response was comparable between obese and lean animals which is in agreement with mice-fed high fat diet, but in contrast to fa/fa Zucker rats, [32]. However, the splenic lymphocyte proliferative response to mitogen and HBsAg upon HBV was reduced in obese animals compared to lean animals. Such discrepancies in response under normal and sensitized conditions were also observed in db/db, ob/ob mice models where no difference was seen in the proliferative activity in vitro but on sensitization the obese animals exhibited decreased proliferative suggesting a deleterious microenvironment in terms of altered hormonal and metabolic status [33, 34]. 

Decreased HBsAg-specific IgG antibody response was however, seen similar to obese human subjects, where there were decreased antibody titers to Hepatitis B vaccine and tetanus toxoid [11–13]. This could be attributed to mechanical factors like insufficient dose relative to body size or suboptimal absorption and distribution of the injected vaccine under obesity [35].

NO generated by macrophages or the antigen-presenting cells (APCs) during the process of antigen presentation to T cells contributes to the control of replication or killing of intracellular microbial pathogens [36]. WNIN/GR-Ob obese unvaccinated rats showed elevated NO production by peritoneal macrophages upon stimulation with LPS compared with their lean unvaccinated animals. These observations are in agreement with the findings observed in db/db mice wherein the heightened NO production was attributed to the hyperglycemia observed in those animals [37]. Though the basal NO levels were high, vaccination did not lead to further increase in NO production in obese, which was however shown by lean littermates. The blunted NO production by macrophages observed in WNIN/GR-Ob obese vaccinated animals may be due to the presence of high basal NO levels in macrophages of unvaccinated obese rats. Elevated NO in macrophage was shown to downregulate protein synthesis, phagocytic activity, respiratory burst, expression of MHC class II, and suppression of transcriptional activation of several genes including nitric oxide synthase 2 and may even trigger their own apoptosis [38]. However, the peritoneal macrophage NO production by euglycemic WNIN/Ob obese unvaccinated and vaccinated animals was unaltered [14].

Tumor Necrosis Factor-*α* (TNF-*α*) secreted by monocytes or macrophages upon Lipopolysaccharide (LPS) stimulation is essential for host defense against bacterial and other pathogens [39]. Furthermore, it enhances the antigen presenting capacity and the T-lymphocyte proliferation [40]. There was no significant difference in the LPS stimulated TNF-*α* release by peritoneal macrophages between obese and lean unvaccinated animals which is in agreement with the findings in diet-induced obese rats [41]. Unlike WNIN/Ob, the LPS stimulated TNF *α* release by peritoneal macrophages was decreased in WNIN/GR-Ob obese vaccinated animals compared to lean vaccinated animals [14].

Earlier studies had shown that the hormonal and metabolic abnormalities associated with obesity such as hyperinsulinemia and hyperlipidemia decreases cellular immune functions such as natural killer cell activity and proliferation of peripheral blood lymphocytes [42–45]. Apart from the these factors, it is also shown that in prediabetic state per se, the lymphomononuclear (LMN) cells change their energy pathway leading to anaerobic glycolysis and this abnormal energy production might cause dysfunction in LMN cells and the immune system in diabetic and prediabetic patients [46]. Furthermore, an association of Hepatitis C virus with insulin resistance among adults was observed and abnormal glucose tolerance was found to be more common in HCV-seropositive individuals than seronegative individuals among obese participants suggesting that it could be an additional risk factor in the impairment of immune function of WNIN/GR-Ob obese rats [47].

## 5. Conclusion

WNIN/GR-Ob obese rat with IGT, a suitable model to study obesity associated prediabetic condition showed impaired or altered adaptive and innate immunity upon HBV. This inability of obese animals to respond to HBV has implications for maintenance of protective immunity and immunological memory following vaccination.

##  Authors' Contribution

P. Bandaru was involved in the animal handling, tissue dissection, flow cytometry, cell culture work, antibody estimation, data analysis, interpretation of data and prepared the first draft. H. Rajkumar and G. Nappanveettil drafted the manuscript, had overall supervision and gave final approval of the manuscript to be published.

## Figures and Tables

**Table 1:** Spleen weight, lymphocyte subsets, lymphocyte proliferative response, and serum IgG and IgM levels in 3-month-old WNIN/GR-Ob lean and obese rats.

Immune parameters	3 month old WNIN/GR-Ob females	
	Lean	Obese
Spleen weight (mg)/g body weight	2 ± 0.07*	1.3 ± 0.05
Total T cells (%)	43.9 ± 1.64*	37.0 ± 1.92
T helper cells (%)	38.1 ± 1.58*	29.1 ± 1.8
T cytotoxic cells (%)	14.7 ± 0.9	16.8 ± 0.48
Total B cells (%)	29.5 ± 3.22	23.4 ± 1.01
Splenic lymphocyte proliferative response (T/C)	11.6 ± 1.95	7.9 ± 1.85
IgG levels (mg/mL)	2.02 ± 0.184*	3.01 ± 0.38
IgM levels (*μ*g/mL)	16.7 ± 1.24*	27.7 ± 2.46

Values are in mean ± SE; *P < 0.05 (significant difference between lean and obese rats).

**Table 2:** Mitogen stimulated IL2 cytokine production by splenocytes and LPS-stimulated TNF-*α* and NO production by peritoneal macrophages to Hepatitis B vaccine in 3-month-old WNIN/GR-Ob lean and obese rats.

Immune parameters	WNIN/GR-Ob lean unvaccinated (*n* = 6)	WNIN/GR-Ob obese unvaccinated (*n* = 6)	WNIN/GR-Ob lean vaccinated (*n* = 8)	WNIN/GR-Ob obese vaccinated (*n* = 8)
Con A stimulated IL2 production (ng/mL)	1505 ± 446	1087 ± 149	1423 ± 323	940 ± 290
LPS stimulated TNF-*α* release (ng/mL)	1642 ± 748^a,b^	430 ± 17^a,b^	1974 ± 449^∗a^	384 ± 28^b^
LPS stimulated NO production (ng/mL)	1.96 ± 0.35^∗a^	4.4 ± 0.35^b^	4.7 ± 0.66^b^	4.25 ± 1.34^a,b^

Values are in mean ± SE; *P < 0.05 (significant difference between unvaccinated and vaccinated groups of lean and obese rats). The means bearing similar superscripts in each row do not differ significantly.
